# Numbers of articles in the three Japanese national newspapers, 1872–2021

**DOI:** 10.1038/s41597-024-03245-9

**Published:** 2024-05-02

**Authors:** Yuji Ogihara

**Affiliations:** 1 Institute of Arts and Sciences, Tokyo University of Science, Tokyo, Japan; 2 Department of Psychology, College of Education, Psychology and Human Studies, Aoyama Gakuin University, Tokyo, Japan

**Keywords:** Social sciences, Psychology, Environmental social sciences

## Abstract

Newspapers have been analyzed in many disciplines, including the humanities, social sciences, and natural sciences. However, previous research using Japanese newspapers investigated the absolute frequency (number) of articles of interest and did not examine the relative frequency (rate) of articles, restricting a deeper understanding of humans, society, and nature. The absolute frequency and the relative frequency of articles can show different patterns of results, which leads to different conclusions. Thus, investigating only the absolute frequency of articles is insufficient, or sometimes misleading. Therefore, it is necessary to examine not only the absolute frequency of articles but also their relative frequency. For this purpose, I conducted a series of systematic searches and provided the yearly numbers of articles in the three databases of Japanese national newspapers over the 150 years between 1872 and 2021. This paper enables researchers to calculate the relative frequency of articles, contributing to research in many disciplines.

## Background & Summary

### Newspapers as an important tool for research

Newspapers have been analyzed in research in many academic disciplines, including the humanities (e.g.^1,2^), social sciences (e.g.^3,4^), and natural sciences (e.g.^5,6^). Analyzing newspapers is a frequently used approach for at least three reasons.

First, newspapers reflect the interests and attentions of people in general, through which researchers can examine humans, society, and nature. Because newspaper companies must sell as many newspapers as possible in a competitive market, writers and editors choose topics and content of articles based on what people in general are interested in and pay attention to at the moment (e.g., recent natural disasters, timely political events). Thus, topics and content are strongly influenced by public interests and attentions.

Second, newspapers are a product that reflects group-level elements of culture (e.g.^7,8^), which is one of the important objects of examination. For example, cultural norms affect contents and topics of articles. Because newspapers have strict space and time constraints, writers and editors must limit an amount of information. In this process, norms affect selections of articles/topics regarding which articles/topics are important and should be included (or excluded).

Third, newspapers are a cultural product that remains over time (for reviews, see^9,10^), which enables researchers to empirically examine changes from the past to the present. Fundamentally, it is difficult to examine historical changes because it is impossible to go back to the past and conduct experiments and surveys. Thus, newspapers are a desirable tool for investigating historical changes. In fact, they have been frequently used to analyze cultural changes (e.g.^11,12^).

### A significant limitation of past research in Japan: relative frequency of articles was not examined

However, most previous research did not examine the relative frequency of articles (rate of articles) of interest. Most studies investigated the absolute frequency of articles (number of articles), restricting a deeper understanding of humans, society, and nature. As far as I looked over, studies that indicated the yearly total number of articles and calculated the rates of articles (dividing the number of articles by the yearly total number of articles) were not found in Japan. At least in most studies, the absolute numbers of articles have been investigated, but the rates of articles have not been commonly investigated.

It is necessary to examine not only the absolute frequency of articles but also their relative frequency. This is because absolute frequency and relative frequency can show different patterns of results, reaching different conclusions. Thus, investigating only absolute numbers of articles is insufficient, or sometimes misleading.

For example, a study found an increase in numbers of newspaper articles mentioning a concept and concluded that society emphasized the concept more strongly over that period. Nevertheless, if the numbers of total articles increased more remarkably than the numbers of articles mentioning the concept, the rates of articles mentioning the concept could decrease. This implies that society de-emphasized the concept over the period, which is opposite to the initial conclusion.

For another instance, a study reported that the numbers of newspaper articles mentioning a concept were stable and concluded that society did not change its emphasis on the concept for the period. Yet, if the number of total articles increased (decreased), the rates of articles mentioning the concept could decrease (increase). This implies that society de-emphasized (emphasized) the concept over the period, which is a totally different conclusion.

Therefore, access to the total yearly numbers of articles in databases enables researchers to calculate the relative frequencies of articles in addition to the absolute frequencies. This contributes to research in many academic disciplines including the humanities, social sciences, and natural sciences because the databases have been commonly used in this wide range of academic fields.

Moreover, this paper becomes archived historical data at present. Numbers of articles in the databases can change over time. Especially for the updates of databases, newspaper companies gradually add new articles to their databases. In contrast, companies sometimes remove previous articles from their databases for some reasons (e.g., infringing copyrights, protecting personal information). Thus, it is important to record information in the databases similar to a time stamp.

### The current paper

The current paper provides the yearly number of articles in the three databases of Japanese national newspapers (the three databases are explained in detail below). To do this, I conducted a series of systematic searches in the databases.

## Methods

### Three databases of the Japanese national newspapers

Three major national newspapers were analyzed: the Yomiuri Shimbun (読売新聞), the Asahi Shimbun (朝日新聞), and the Mainichi Shimbun (毎日新聞) (“Shimbun” means newspaper in Japanese). These newspapers have been the most popular national newspapers in Japan (the big three newspapers): the Yomiuri Shimbun was the bestselling newspaper in Japan. the Asahi Shimbun was second, and the Mainichi Shimbun was third^13^.

These newspapers have been popular not only in Japan but also worldwide. In fact, in the ranking of world daily newspapers in circulation in 2015, the Yomiuri Shimbun was first, the Asahi Shimbun was second, and the Mainichi Shimbun was sixth (^14^; also see^15^). Furthermore, the Yomiuri Shimbun has the world record for the largest daily circulation in the Guinness Book of World Records (13,537,276 issues distributed in 2010^16^).

These Japanese newspaper companies offer systematic online databases. Thus, I used these databases of each newspaper: Yomidas Rekishikan (ヨミダス歴史館; the database of the Yomiuri Shimbun), Kikuzo II Visual (聞蔵IIビジュアル; the database of the Asahi Shimbun; The name of this database changed in April 2022. The new and current version of the name is Asahi Shimbun Cross-Search. Contents of the database did not change due to the change of the name. The present article focuses on the articles until 2021 before the name was changed, so the previous name, Kikuzo II Visual, is used in this article), and Maisaku (毎索; the database of the Mainichi Shimbun). A summary of these three databases is indicated in Table 1.

**Table 1:** Summary of the three databases of the Japanese national newspapers.

	Yomidas Rekishikan (ヨミダス歴史館)	Kikuzo II Visual (聞蔵IIビジュアル)	Maisaku (毎索)
Newspaper	The Yomiuri Shimbun	The Asahi Shimbun	The Mainichi Shimbun
Period	1874–2021	1879–2021	1872–2021
Years	148 years	143 years	150 years
Datasets	Scanned image (1874–1989; 116 years)	Scanned image (1879–1999; 121 years)	Scanned image (1872–1986; 115 years)
	Text (1986–2021; 36 years)	Text (1984–2021; 38 years)	Text (1987–2021; 35 years)
Number of articles	Scanned image (1874–1989): 4,539,324	Scanned image (1879–1999): 5,761,309	Scanned image (1872–1986): 2,325,658
	Text (1986–2021): 8,424,760	Text (1984–2021): 9,103,980	Text (1987–2021): 7,434,078
	Total: 12,964,084	Total: 14,865,289	Total: 9,759,736

Each of these databases consists of two parts: scanned image and text. Older newspapers are archived as images. The articles in this part are stored with text headings. Users can search for articles using these headings (contents are not searchable). Newer newspapers are archived as text. Articles in these newspapers are stored with texts. Thus, users can search for articles both by their content and by their headings.

#### Yomidas Rekishikan (ヨミダス歴史館; the database of the Yomiuri Shimbun)

Newspapers between 1874 and 1989 (116 years) are archived as images. The inclusion of articles in 1874 started in November, which means that the number of articles in 1874 covers two months. This former part had 4,539,324 articles in total. Newspapers between 1986 and 2021 (36 years) are archived as text. The inclusion of articles in 1986 started in September, meaning that the number of articles in 1986 covers four months. This latter part had 8,424,760 articles in total. Thus, the total number of articles that this database included was 12,964,084.

#### Kikuzo II Visual (聞蔵IIビジュアル; the database of the Asahi Shimbun)

Newspapers between 1879 and 1999 (121 years) are archived as images. This part had 5,761,309 articles. Newspapers between 1984 and 2021 (38 years) are archived as text. This part had 9,103,980 articles. Thus, the total number of articles was 14,865,289.

#### Maisaku (毎索; the database of the Mainichi Shimbun)

Newspapers between 1872 and 1986 (115 years) are archived as images. The major articles in this part are stored with headings. This part had 2,325,658 articles. Newspapers between 1987 and 2021 (35 years) are archived as text. This part had 7,434,078 articles. Thus, the total number of articles was 9,759,736.

### Procedure

To obtain the number of articles in the databases by year, I conducted a series of searches without entering words in the search box in each of the databases for a given year. Usually, words or phrases are entered to search articles, but here, I intentionally entered no words in the search box.

## Data Records

**Fig. 1:**
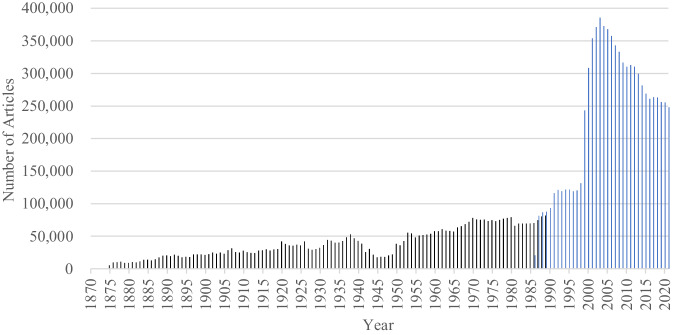
The numbers of articles in the Yomidas Rekishikan (ヨミダス歴史館; the database of the Yomiuri Shimbun; 1874–2021). *Note*. Black bars indicate the numbers of articles in the scanned image dataset (1874–1989), and blue bars indicate the numbers of articles in the text dataset (1986–2021).

**Fig. 2:**
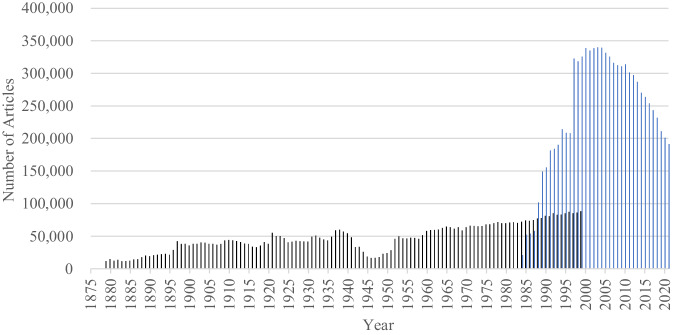
The numbers of articles in the Kikuzo II Visual (聞蔵IIビジュアル; the database of the Asahi Shimbun; 1879–2021). *Note*. Black bars indicate the numbers of articles in the scanned image dataset (1879–1999), and blue bars indicate the numbers of articles in the text dataset (1984–2021).

**Fig. 3:**
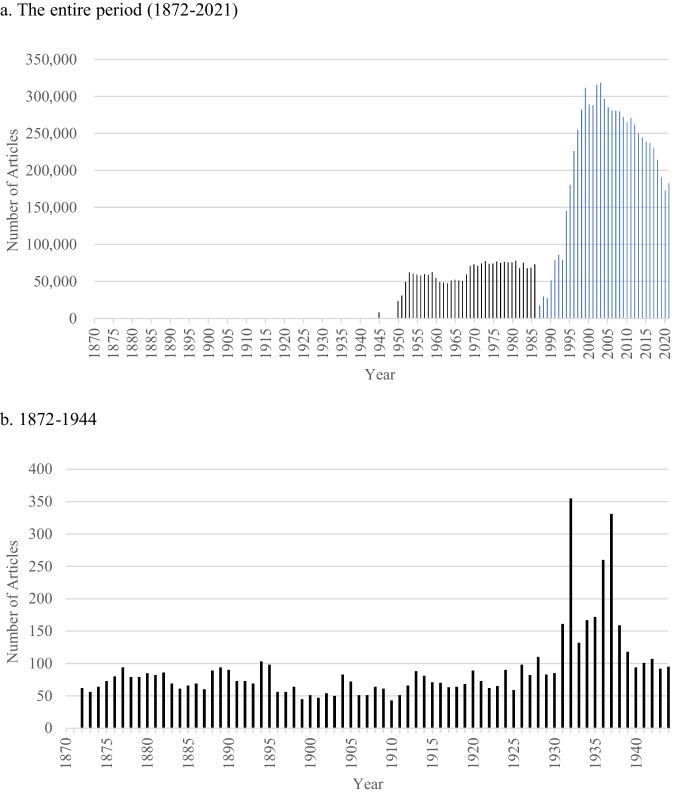
The numbers of articles in the Maisaku (毎索; the database of the Mainichi Shimbun; 1872–2021). *Note*. Black bars indicate the numbers of articles in the scanned image dataset (1872–1986), and blue bars indicate the numbers of articles in the text dataset (1987–2021).

Performing these procedures, the numbers of articles in each of the three national newspapers by year are indicated in Fig. 1 (Yomidas Rekishikan; ヨミダス歴史館; the database of the Yomiuri Shimbun), Fig. 2 (Kikuzo II Visual; 聞蔵IIビジュアル; the database of the Asahi Shimbun), and Fig. 3 (Maisaku; 毎索; the database of the Mainichi Shimbun). The raw data are archived on the Open Science Framework (OSF) platform (10.17605/OSF.IO/F8SH3^17^).

In Fig. 3a, which visualizes the numbers of articles between 1872 and 2021, the numbers of yearly articles between 1872 and 1944 are difficult to see because they are relatively smaller than those after the period. Thus, I added the other figure (Fig. 3b) focusing on this period.

**Fig. 4:**
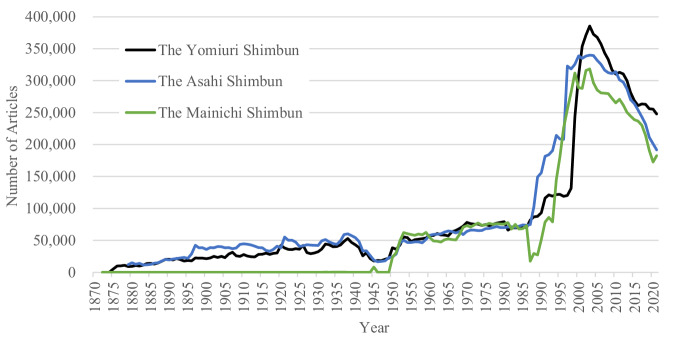
The numbers of articles in the three newspaper databases (1872–2021). *Note*. In the periods when there were two values from both the scanned image dataset and text dataset in the Yomidas Rekishikan (the Yomiuri Shimbun database) and the Kikuzo II Visual (the Asahi Shimbun database), larger values were used.

Figure 4 shows the numbers of articles in all three national newspapers by year to see differences among the three newspapers.

In this article, I provided the total numbers of articles by year in the databases. By applying this procedure, numbers of articles by component, such as sections (e.g., politics, economic), regions (e.g., Tokyo, Osaka), and time periods (e.g., before and after World War II, during major natural disasters) are also available.

## Technical Validation

It is necessary to confirm whether the procedure indeed captures the number of articles by year in the databases: the validity of this procedure. I asked each of the newspaper companies (the Yomiuri Shimbun, the Asahi Shimbun, and the Mainichi Shimbun) whether a search without entering words in the search box indeed yields the number of articles for a given year. All the companies answered that this assumption is correct. Thus, the validity of this procedure has been officially confirmed.

## Usage Notes

Three notes should be explained to use these data appropriately. First, these numbers are not equal to the numbers of articles published in printed versions of the newspapers. They might be different from each other. The databases do not include some articles for some reasons such as infringement of copyrights and protection of private information.

Second, the numbers in the databases are at the point of December 2022. As explained above, these numbers can change over time. Thus, if users of these datasets need accurate numbers of articles at a given time, it is recommended that they follow the same procedure that I explained above. If users do not need very exact numbers of articles, they can use these datasets as they are.

Third, the numbers do not include the number of advertisements published in newspapers because they are different from written articles in nature.

## Data Availability

No code was developed for this work.
